# Increased Serum ox-LDL Levels Correlated with Lung Function, Inflammation, and Oxidative Stress in COPD

**DOI:** 10.1155/2013/972347

**Published:** 2013-09-01

**Authors:** Yongchun Shen, Ting Yang, Shujin Guo, Xiao'ou Li, Lei Chen, Tao Wang, Fuqiang Wen

**Affiliations:** Division of Pulmonary Diseases, State Key Laboratory of Biotherapy of China and Department of Respiratory and Critical Care Medicine, West China Hospital of Sichuan University, Chengdu 610041, China

## Abstract

Chronic obstructive pulmonary disease (COPD) is associated with abnormal inflammation and high oxidative stress. Studies suggest that oxidized low density lipoprotein (ox-LDL) is involved in diseases associated with oxidative stress and inflammation. However, no data on the possible relationship between COPD and ox-LDL are available. This study compared serum levels of ox-LDL in 48 COPD patients and 32 health controls and correlated them with lung function, systematic inflammation, and oxidative stress. Serum levels of ox-LDL, C-reactive protein (CRP), and oxidative stress (measured by reactive oxygen species, ROS) were analyzed using commercial kits. Mean levels of serum ox-LDL were significantly higher in COPD patients than in controls (18.62 ± 7.56 versus 12.57 ± 5.90 mU/L, *P* < 0.05). Serum levels of CRP and ROS were also significantly higher in COPD patients. Serum levels of ox-LDL in COPD patients correlated inversely with FEV_1_% predicted, an index of lung function (*r* = −0.347, *P* = 0.016), while they correlated positively with CRP and ROS levels. These results suggest that serum levels of ox-LDL are increased in COPD patients and that these levels are associated with lung function, inflammation, and oxidative stress in COPD. Future studies are needed to determine whether and how ox-LDL plays a role in COPD.

## 1. Introduction

Chronic obstructive pulmonary disease (COPD) remains a worldwide health care burden. Currently the fourth leading cause of death, COPD, is projected to become the third leading cause by 2030 [1, 2]. This disease is characterized by progressive, partially reversible airflow obstruction associated with abnormal airway inflammation and oxidant/antioxidant imbalance [3]. Recent studies suggest that in a subset of COPD patients, the inflammation may also “spread” into the circulatory system and cause systematic inflammatory injuries and organ damage [4, 5]. Thus, COPD can no longer be considered a disease only of the lungs. Instead, increased systemic inflammation may contribute to complex chronic comorbidities often observed in COPD patients, such as coronary artery disease, which is associated with poor clinical outcomes in COPD [6]. Despite the seriousness of COPD and its comorbidities, how systemic inflammation occurs in patients is unclear.

A possible explanation for the systemic inflammation in COPD is oxidized low density lipoprotein (ox-LDL), a marker of oxidative stress that links the immune system with the abnormal inflammation. This lipoprotein has a proinflammatory effect, and it enhances inflammatory cytokine production, as well as cell adhesion, migration, proliferation, and apoptosis [7]. Circulating ox-LDL is elevated in several diseases associated with high oxidative stress and inflammation, such as arteriosclerosis and sepsis [8, 9]. Numerous in vitro and animal studies, as well as epidemiological and correlational studies in humans, have proposed that ox-LDL may contribute to atherosclerosis. These findings raise the question of whether COPD, which is characterized by systemic inflammation and high risk of arteriosclerosis [4, 5], is also associated with elevated ox-LDL levels. If so, it may mean that ox-LDL may play a role in the pathophysiology of the disease. 

Despite the importance of these questions, we are unaware of any studies examining a possible relationship between COPD and ox-LDL. Therefore, we carried out a preliminary clinical study to determine whether a relationship exists between COPD and ox-LDL.

## 2. Patients and Methods

### 2.1. Study Protocol

The study protocol conforms to the principles of the Declaration of Helsinki and was approved by the Institutional Review Board for Human Studies of West China Hospital of Sichuan University, China. Written consent was obtained from all subjects. Between October 2012 and January 2013, patients with COPD were recruited from the Outpatient Department of West China Hospital, and healthy controls were recruited from the hospital's physical examination center. All subjects underwent a standard lung function test, and COPD was diagnosed prospectively for this study on the basis of Global Initiative for Chronic Obstructive Lung Disease criteria [1]. 

To be included in the study, patients had to show (a) a ratio of forced expiratory volume in the first second to forced vital capacity (FEV_1_/FVC) below 70% after bronchodilation and (b) an increase in FEV_1_ below 12% after inhalation of *β*2-agonist (200 mg salbutamol), and they had to have remained (c) clinically stable for at least 3 months prior to the study. No COPD patients had received standard COPD treatments, such as inhaled corticosteroids. Patients were excluded if they had conditions known to affect serum levels of ox-LDL, including coronary heart disease and metabolic syndrome, the presence of which was determined based on medical history and fasting blood-glucose tests.

### 2.2. Measurement of ox-LDL, CRP, and ROS

Subjects were asked to fast overnight from 21:00 the night before, after which venous blood samples were collected and serum was separated immediately and stored at −80°C until analysis. Levels of ox-LDL and C-reactive protein (CRP) were analyzed using an enzyme-linked immunosorbent assay (ELISA; Xitang Bio-Technology Co., Ltd., Shanghai, China), and oxidative stress was assessed by measuring levels of reactive oxygen species (ROS) using a colorimetric kit (Xitang Bio-Technology Co., Ltd., Shanghai, China). The ROS kit measures OH^•^ and other free radicals in the serum. All the measurements were carried out strictly according to the manufacturer's instructions. Manufacturer-specified limits of detection were 1 mU/L for ox-LDL, 0.3 ng/mL for CRP, and 15 nmol/mL for ROS. The other biochemical tests, including assays for high-density lipoprotein (HDL), low-density lipoprotein (LDL), triglyceride (TG), total cholesterol (TC), and other indices, were carried out by the Department of Laboratory Medicine of West China Hospital. Technicians performing tests were blinded to the clinical details of the subjects.

### 2.3. Statistical Analysis

Results are presented as mean ± standard deviation (SD). Differences between groups were statistically analyzed using an unpaired Student's *t*-test after confirming that data were normally distributed. Correlations were performed using the bivariate Pearson's correlation test. The threshold of significance was set at 5%. Data were analyzed using SPSS 18.0 for Windows (IBM, Chicago, IL, USA).

## 3. Results

### 3.1. Clinical Characteristics of Subjects

A total of 48 COPD patients and 32 healthy controls participated in this study.  Table 1 summarizes their demographic and clinical characteristics, smoking pack-years, and serum levels of TG, TC, HDL, LDL, ox-LDL, CRP, and ROS. Patients and controls were similar in terms of age, sex, and smoking pack-years. FEV_1_% predicted among COPD patients was 86.87 ± 21.64%, suggesting that most had mild airflow limitation. 

### 3.2. Serum Levels of ox-LDL, CRP, and ROS

**Figure 1:**
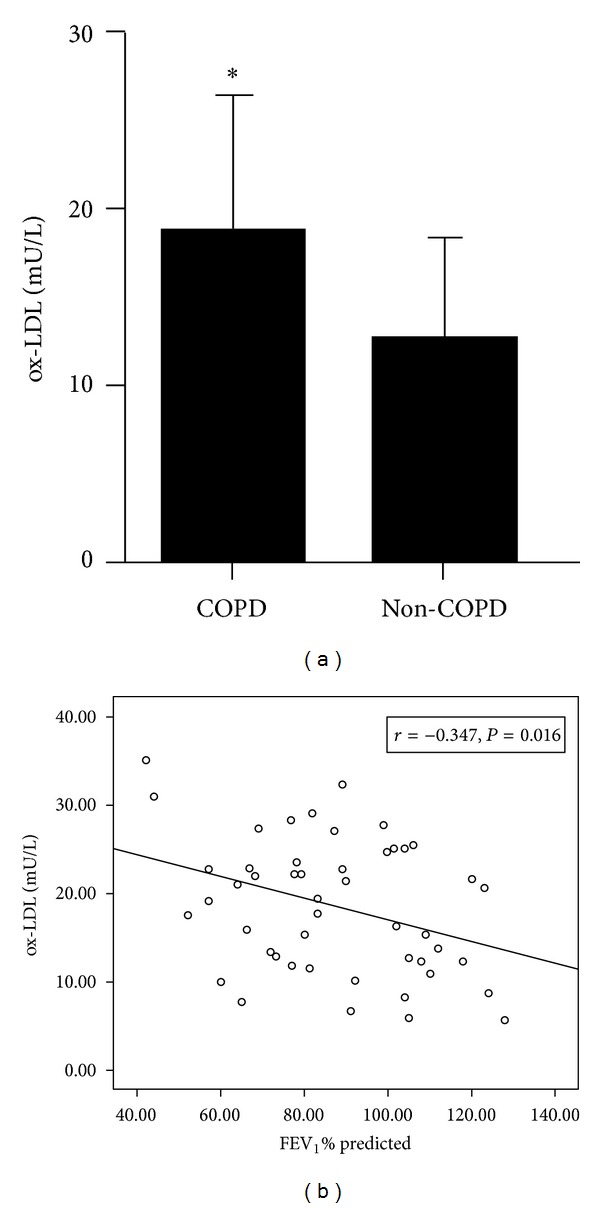
Serum levels of ox-LDL and correlation with lung function. (a) Levels of ox-LDL in COPD patients and healthy controls (*, *P* < 0.05). (b) Correlation between serum ox-LDL levels and lung function (FEV_1_% predicted) in patients.

Mean serum levels of ox-LDL were significantly higher in COPD patients (18.62 ± 7.56 mU/L) than in controls (12.57 ± 5.90 mU/L, *P* = 0.000; Figure 1(a)). The same was true of serum levels of CRP, a marker of systemic inflammation (18.49 ± 5.79 versus 15.21 ± 4.91 ng/mL, *P* = 0.01), and serum levels of ROS, a marker of oxidative stress (293.11 ± 90.44 versus 242.86 ± 70.15 nmol/mL, *P* = 0.01). 

### 3.3. Correlations between ox-LDL and Indicators of Lung Function, Inflammation, and Oxidative Stress

**Figure 2:**
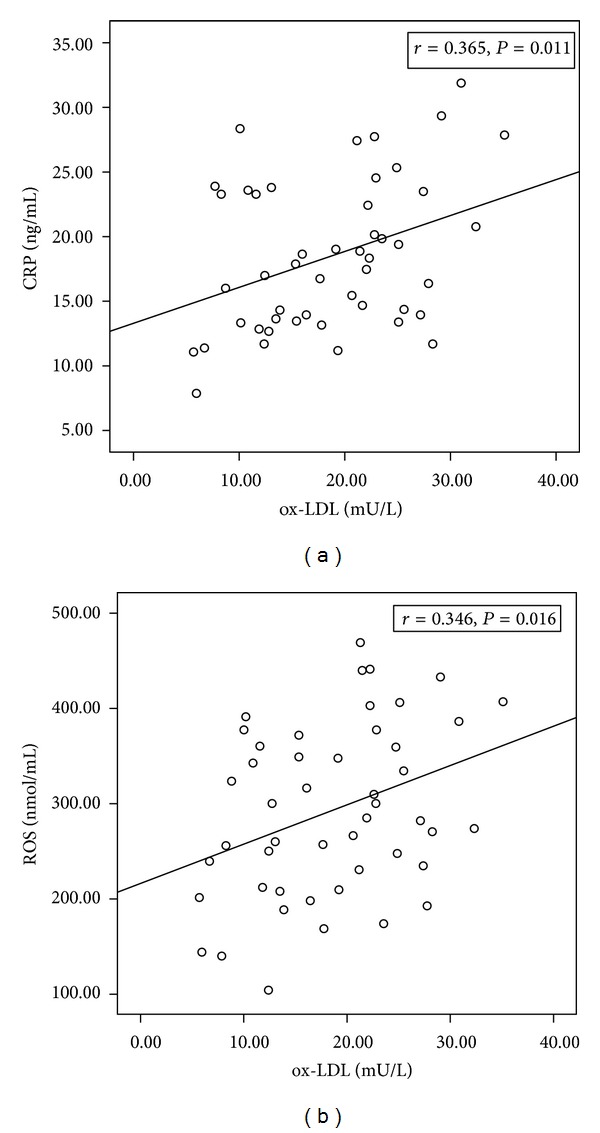
Correlations between serum levels of ox-LDL and (a) CRP or (b) ROS in COPD patients.

Serum levels of ox-LDL in COPD patients correlated inversely with lung function, based on FEV_1_% predicted (*r* = −0.347, *P* = 0.016; Figure 1(b)). In addition, serum levels of ox-LDL in patients also correlated inversely with the ratio of FEV_1_/FVC (*r* = −0.412, *P* = 0.004). Conversely, ox-LDL levels in patients correlated positively with CRP (*r* = 0.365, *P* = 0.011; Figure 2(a)) and ROS (*r* = 0.346, *P* = 0.016; Figure 2(b)). 

## 4. Discussion

To our knowledge, this is the first study to examine correlations between serum levels of ox-LDL levels in COPD patients and indicators of lung function, inflammation, and oxidative stress. We found significant correlations between ox-LDL levels and lung function decline (measured as FEV_1_% predicted), inflammation (CRP), and oxidative stress (ROS). These findings justify further detailed work into the mechanistic basis of these correlations in order to determine whether and how ox-LDL levels contribute to the pathogenesis of COPD.

Circulating ox-LDL has been proposed to play a critical role in cardiovascular diseases such as atherosclerosis and to help upregulate the inflammatory response. Our findings suggest that it may also play a role in COPD. Such a role would be consistent with numerous lines of indirect evidence linking ox-LDL with this disease. First, ox-LDL activates several transcription factors involved in the pathogenesis of COPD, including activator protein 1, NF-*κ*B, signal transducer and activator of transcription (STAT), and hypoxia-inducible factor 1 [10]. Second, an increase in ox-LDL levels activates chemotaxis of neutrophil or eosinophil granulocytes and monocytes in studies in vitro designed to mimic worsening airway inflammation [8, 11]. Third, ox-LDL has been shown to increase the expression or secretion of chemokines such as monocyte chemotactic protein-1, macrophage inflammatory protein- (MIF-)1*α*, and MIF-2, as well as pro-inflammatory cytokines such as interleukin (IL)-1*β*, IL-12, and tumor necrosis factor-*α* [7, 12]. All these inflammatory mediators are known to contribute to COPD pathogenesis. Fourth, oxidized lipids are thought to promote a shift from acute to chronic inflammation [13], and this transition is a key characteristic of COPD. Fifth, ox-LDL increases intracellular ROS production [14], thereby increasing oxidative stress and potentially activating stress kinases and redox-sensitive transcription factors that enhance inflammation. This signaling increases the expression of a battery of distinct pro-inflammatory mediators that contribute to COPD [3]. Indeed, our laboratory has shown that ox-LDL up-regulates TGF-*β*1 production, Smad3 phosphorylation, and Ras/extracellular signal-regulated kinase activity in a dose- and time-dependent manner in A549 human alveolar epithelial cells [15]. These signaling molecules are known to be involved in the development of COPD. The present study extends the literature by showing that serum levels of ox-LDL are increased in COPD, suggesting for the first time that ox-LDL may be involved in the disease.

Our study also identified elevated serum levels of CRP and ROS in COPD patients relative to controls, which is consistent with previous studies [16, 17]. Circulating CRP levels are elevated in COPD patients and may thus be regarded as a valid biomarker of systemic inflammation [17]; in addition, serum CRP levels are associated with FEV_1_ decline [18]. These results suggest that CRP and ROS may contribute to the chronic inflammation and oxidative stress characteristic of COPD. Another important observation in our study is the inverse correlation between ox-LDL and lung function (FEV_1_% predicted) in patients, suggesting that ox-LDL may correlate with the severity of airway obstruction. In addition, ox-LDL in patients correlated positively with CRP and ROS, suggesting that ox-LDL may function as a biomarker of the severity of systematic inflammation and oxidative stress in this disease. Future studies should examine how ox-LDL is produced in COPD patients and what signaling pathways it triggers. Previous work suggests that smoking over the clinical course of COPD may lead to the production of ox-LDL [19], but our two groups showed similar levels of smoking. Larger, more detailed studies are needed to clarify whether and how smoking may contribute to ox-LDL levels and to COPD in general.

COPD exerts significant extrapulmonary effects that may contribute to its severity. A major comorbidity of COPD is coronary heart disease (also called atherosclerotic heart disease), which is the leading cause of death in patients with the disease [20]. Why these two diseases are so frequently found in combination remains unclear. Studies have implicated systematic inflammation and oxidative stress as pathophysiological links between COPD and coronary heart disease [21]. We speculate that the elevated serum levels of ox-LDL in COPD patients not only induce inflammation and oxidative stress but also play a role in promoting arteriosclerosis. In this way, ox-LDL may function as a bridge between COPD and coronary heart disease. 

The primary goal of this study was to determine whether levels of ox-LDL correlate with COPD. While we achieved this goal by showing that there is indeed a correlation, the fact that we used a limited number of subjects and only two indicators to analyze systemic inflammation (CRP) and oxidative stress (ROS) means that we cannot gain detailed insights into whether and how ox-LDL contributes to the disease. Our small number of subjects also means that we cannot definitively conclude whether smoking contributed or not to our results. Given the strong evidence for smoking as a risk factor for COPD, larger studies specifically designed to assess the role of smoking in COPD-related inflammation and oxidative stress are needed. Furthermore, most of our patients presented with relatively mild COPD, so it would be important to verify our findings in patients with more severe disease. 

To avoid the limitations of the present work, future studies should use computed tomography to detect the presence of emphysema, since our results may be biased by the relative numbers of subjects with “blue bloater” versus “pink puffer” phenotypes [22]. Future studies should also examine additional markers of systemic inflammation and oxidative stress in order to gain a more detailed and reliable picture of the role of ox-LDL in COPD. Given that ox-LDL is known to be a risk factor for coronary heart disease, subjects should be analyzed using echocardiography in order to correlate cardiac and lung function. 

## 5. Conclusion

In this prospective study, serum levels of ox-LDL were higher in COPD patients than in healthy controls. Levels of ox-LDL correlated inversely with lung function and positively with serum levels of CRP and ROS. Larger, more detailed studies are needed to confirm our findings and determine whether and how high ox-LDL levels play a role in COPD.

## Figures and Tables

**Table 1:** Characteristics of patients with COPD and healthy subjects.

Characteristic	COPD (*n* = 48)	Control (*n* = 32)	*P* value
Age (year)	62 ± 10	58 ± 11	0.177
Sex (m/f)	33/15	22/10	0.626*
BMI (kg/m^2^)	22.57 ± 3.25	23.04 ± 2.62	0.494
TG (mmol/L)	1.12 ± 0.46	1.31 ± 0.45	0.071
TC (mmol/L)	4.66 ± 0.73	4.81 ± 0.78	0.376
HDL (mmol/L)	1.60 ± 0.31	1.55 ± 0.37	0.489
LDL (mmol/L)	2.75 ± 0.58	2.51 ± 0.59	0.078
Smoking (pack years)	13.93 ± 16.34	12.08 ± 19.69	0.457
FEV_1_ (L)	2.06 ± 0.78	2.66 ± 0.61	0.000
FVC (L)	3.40 ± 1.13	3.21 ± 0.77	0.367
FEV_1_/FVC%	59.73 ± 8.19	83.30 ± 5.05	0.000
FEV_1_% predicted	86.87 ± 21.64	115.22 ± 15.91	0.000
oxLDL (mU/L)	18.62 ± 7.56	12.57 ± 5.90	0.000
CRP (ng/mL)	18.49 ± 5.79	15.21 ± 4.91	0.01
ROS (nmol/mL)	293.11 ± 90.44	242.86 ± 70.15	0.01

Data are presented as mean ± SD. *The chi-squared test was used to test the significance of the difference in gender proportions.

Abbreviations: COPD: chronic obstructive pulmonary disease; CRP: C-reactive protein; FEV_1_: forced expiratory volume in one second; FVC: forced vital capacity; HDL: high-density lipoprotein; LDL: low-density lipoprotein; ox-LDL: oxidized low-density lipoprotein; ROS: reactive oxygen species; TC: total cholesterol; TG: triglyceride.
